# Exploring the smoking cessation needs of individuals with diabetes using the Information-Motivation-Behavior Skills model

**DOI:** 10.18332/tpc/181366

**Published:** 2024-02-02

**Authors:** Joseph Grech, Ian J. Norman, Roberta Sammut

**Affiliations:** 1 Department of Nursing, Faculty of Health Sciences, University of Malta, Msida, Malta; 2 Faculty of Nursing, Midwifery and Palliative Care, King’s College London, United Kingdom

**Keywords:** diabetes mellitus, diabetes complications, tobacco use cessation, smoking cessation agents, counselling, information motivation behavioral skills model

## Abstract

**INTRODUCTION:**

Smoking cessation is an important aspect of diabetes management. Despite the increased risk for diabetes complications when smoking, evidence suggests that people living with type 1 and type 2 diabetes are less likely to quit smoking when compared to those without diabetes. Guided by the Information-Motivation-Behavioral Skills model, this study aimed to identify the needs of individuals living with type 1 and type 2 diabetes to quit smoking.

**METHODS:**

A qualitative descriptive design was adopted. Semi-structured telephone interviews were held between April and June 2021, with 20 former and current Maltese smokers living with type 1 or type 2 diabetes, recruited from the diabetic clinics within the two main acute public hospitals. The interview transcriptions were analyzed using applied thematic analysis.

**RESULTS:**

Individuals with diabetes need more information on the effects of smoking on diabetes to encourage cessation. Preventing diabetic complications was reported as a motivator to quit smoking. However, having diabetes was identified as a challenge to quitting. Participants welcomed the provision of health professional support for quitting smoking, identifying the need to provide smoking cessation support within diabetic clinics. The provision of information on tobacco-associated diabetic complications, by using video messages featuring former smokers’ stories was also suggested.

**CONCLUSIONS:**

To promote smoking cessation among individuals with diabetes, they need to be informed about how smoking affects their condition. Utilizing video messages featuring real-life stories of former smokers with diabetes who experienced tobacco-associated diabetic complications may be influential. Additionally, providing diabetes-specific intensive smoking cessation support is crucial to help them quit.

## INTRODUCTION

Diabetes mellitus is a global epidemic affecting an estimated 537 million people worldwide^1^. Diabetes has also been recognized as a public health priority in Malta^2^. Although when compared to the other World regions, the European region has the second-lowest diabetes prevalence (9.2%; 95% CI: 7.1–10.4), Malta, a European country, has a high diabetes prevalence, estimated at 11.2% (95% CI: 8.7–13.8)^1^.

People living with type 1 and type 2 diabetes require medical care, self-management education, and support that goes beyond glucose management^3^. Smoking cessation (and prevention) is an important aspect of diabetes management ^4,5^. Smoking worsens the cardiometabolic parameters of both individuals with type 1 and type 2 diabetes^6^, increasing the risk of diabetes-related complications, such as coronary heart disease, stroke, heart failure, peripheral arterial disease, and even death^7^. Conversely, smoking cessation is associated with better cardiometabolic values^6^ and reduced risks of cardiovascular morbidity and mortality^8^. Smoking cessation for individuals with diabetes is one of the key recommendations of Malta’s national diabetes strategy^2^.

Despite the benefits of quitting, evidence suggests that individuals living with diabetes are less likely to quit smoking when compared to those without diabetes^9,10^. Several diabetes-related factors may hinder the quitting process. Factors such as depression^11^ or physical suffering^12^ caused by diabetes, have been identified by individuals with diabetes as barriers to quitting. Weight gain on cessation, which may lead to poor glycemic control, is also a common concern for patients with diabetes in attempting to quit smoking^13^. Individuals with diabetes also tend to believe that smoking helps them manage diabetes, such as in glycemic control, adherence to diet, or weight management, making them reluctant to stop^12,13^. In view of these diabetes-specific barriers and challenges to quitting, the provision of tailored smoking cessation support has been recommended for individuals with diabetes^5,10^.

Healthcare interventions, such as smoking cessation interventions, rely heavily on patient involvement and their attitudes toward the intervention^14^. Consequently, there is a need to explore recipients’ perspectives on the proposed features of an intervention, as well as other needs that may not have been identified in the literature. This approach has been recommended in recent guidelines for the development of tailored healthcare interventions^15,16^. The Information-Motivation-Behavioral Skills (IMB) model by Fisher et al.^17^ can help in identifying the unique needs of individuals with diabetes to quit smoking, and supporting the development of smoking cessation interventions^18^. The IMB model asserts that behavior change happens when individuals are well-informed, highly motivated, and possess the necessary skills to perform the required behavior change^17^. By understanding the specific IMB factors that are relevant to the particular health behavior and target population, researchers can promote behavior change through tailored interventions^17,18^.

In developing an IMB-based intervention, one needs to first identify the specific IMB factors that are relevant to the particular health behavior and to the target population, through elicitation research^17^. This helps to identify the population-specific IMB strengths, which can be capitalized on, and any deficits that need to be addressed when designing the population-specific intervention^17,18^. Qualitative research may also help to identify challenges and barriers to behavior change, that is, any situational and individual characteristics that can negatively influence the desired behavior change; these may act as moderating factors^17^. Negative moderators, present at high or intense levels, may impinge on the intervention’s effectiveness, thus necessitating change to the proposed intervention or an adjunct effort^17^. The IMB model also asserts that individual objective and subjective health outcomes (e.g. poor glycemic control on quitting) can also act as moderators, as they are directly linked with adherence to the desired behavior change^17^. These, in turn, can influence behavior change via a feedback loop that affects the IMB constructs, strengthening or weakening adherence^17^.

The IMB model has been widely utilized to understand the behavior mechanisms that need to be altered to achieve and sustain behavior change^18^. In exploring diabetes self-care-related IMB factors, Osborn et al.^19^ were able to tailor a diabetes self-care intervention for Puerto Ricans with type 2 diabetes, effectively improving food label reading, diet adherence, and glycemic control at three months follow-up. The IMB model has also been found to be a useful framework for identifying the unique needs of opiate-dependent smokers^20^ and smokers living with HIV^21^ for the development of feasible and acceptable smoking cessation interventions. Guided by the IMB model, Georges et al.^12^ explored the association between diabetes, smoking, and gender, to develop a gender and diabetes-specific smoking cessation intervention^12^. However, they only included individuals with type 2 diabetes in their study and limited their analysis to the experiences of current smokers^12^. Given that individuals with type 1 diabetes and former smokers with diabetes were not represented in the study of Georges et al.^12^, and considering that none of the studies identified in a recent scoping review on smoking cessation and diabetes explored the perspectives of individuals with diabetes on evidence-based smoking cessation recommendations^22^, further research was warranted.

This study aimed to identify the unique needs of individuals with type 1 and type 2 diabetes to quit smoking, for the future development of a tailored smoking cessation intervention. This study explored the smoking cessation-related IMB factors among Maltese individuals with diabetes and their views of the features of smoking cessation interventions, previously identified in a scoping and a systematic review as showing promise in use with persons with diabetes^22,23^. The features of the smoking cessation interventions identified included the provision of intensive professional smoking cessation support, the use of pharmacotherapy for smoking cessation, and the provision of information on tobacco-associated diabetic complications, by using visual images and/or video messages featuring former smokers who experienced tobacco-associated diabetic complications.

## METHODS

### Design

A qualitative descriptive design was utilized. The use of qualitative descriptive research has been recommended for exploring recipients’ views of a proposed intervention or its features, and for identifying other needs as part of the developmental process of healthcare interventions^16^.

The IMB model by Fisher et al.^17^ was used as a guiding framework to identify the unique needs of individuals with type 1 and type 2 diabetes to quit smoking, for the future development of a tailored smoking cessation intervention. Thus, this research looked into identifying the diabetes-specific IMB strengths and any deficits, as well as the challenges and barriers to smoking cessation (negative moderating factors)^17^. Furthermore, considering that both specific objective and subjective health outcomes (e.g. poor glycemic control upon quitting) can function as moderators, either strengthening or weakening adherence to the new behavior (i.e. smoking abstinence)^17^, these aspects were also explored.

### Participants

Both former and current smokers with type 1 or type 2 diabetes who had tried to quit following a diabetes diagnosis and were able to converse in English or Maltese were eligible for inclusion in this study. Individuals with diabetes who had not attempted to quit smoking following their diabetes diagnosis were excluded. Healthcare professionals working within the Maltese diabetic out-patient clinics within community health centers and at the two main acute public hospitals, family doctors and the Malta Diabetes Association, were invited to help identify interested participants, forwarding their contact details to the research team for recruitment with the patient’s consent.

The sample size was based on the principle of ‘data saturation’^24^, seeing saturation when new data collected repeats what was expressed in the previously collected data^25^. The aim was to ensure that the data collected was sufficient enough to answer the set objectives^24^. In estimating the required sample size, reference was made to the seminal study by Guest et al.^26^, in which saturation was relatively achieved after 12 interviews, and the study by Georges et al.^12^, who achieved saturation after ten individuals and five focus group interviews (15 units of analysis). Thus, it was estimated that data saturation would be achieved after 15 interviews.

### Data collection

This study was carried out during the peak of the second wave of the COVID-19 pandemic. Initially, focus group interviews were the preferred method of data collection. However, following the introduction of new COVID-19 restrictions just prior to the data collection period, which limited public meetings to groups of two, this was changed to individual semi-structured interviews. Given the hesitancy of some participants to meet in-person due to the pandemic situation at that time, these were held over the phone.

Interviews followed a question-and-probe guide, which included questions on personal characteristics and questions based on the IMB model, also addressing the participants’ views of the identified promising smoking cessation components identified by Grech et al.^22,23^. The instrument was translated into Maltese by a professional bilingual translator and back-translated to English (by another bilingual translator) to ensure accuracy. Initially, participants were asked about their personal characteristics, i.e. their sociodemographic characteristics and diabetes and smoking profiles. Then, participants were asked about their knowledge about the harms, risks, and interactions between smoking, smoking cessation, and diabetes, and the information they believed they needed to quit smoking (Information). They were also asked for their views on the provision of information on tobacco-associated diabetic complications that influence smoking habits. This included the use of visual images of tobacco-associated diabetic complications and video messages featuring former smokers who experienced tobacco-associated diabetic complications. Furthermore, participants were asked about their motivational factors to quit smoking and avoid relapse (Motivation), their perceived facilitators to quit smoking, their views on the use of pharmacotherapy for smoking cessation, as well as their opinions on health professional smoking cessation support (Behavioral skills). Participants were also asked about their perceived barriers and challenges to quitting smoking to help identify any characteristics that could negatively impact smoking cessation.

### Procedure

Data were collected between April and June 2021. On indicating their interest in participating in the study, prospective participants were verbally briefed on the purpose of the study and the data-collecting procedure, answering any queries that they had. They were also provided with a detailed information letter and a consent form to sign. Participants were reminded that participation was voluntary and that they were free to withdraw from the study at any time without the need to provide a reason. Participants were assured that refusing to participate or withdrawing from the study did not have any effect on their care whatsoever.

All participants were recruited from the diabetic clinics within the two main acute public hospitals in Malta. Participants were recruited with the aim of achieving data saturation. However, the research team also liaised with the recruitment intermediaries to ensure adequate representation by sex, age, type of diabetes, and smoking status. In total, 20 interviews were held. These took 30–40 minutes each and were held in Maltese or English, depending on the preference of the interviewee. All interviews were moderated by JG, who followed the interview guide.

Before starting the phone interviews, the researcher reminded participants that discussions were confidential and that the data would be rendered anonymous. Participants were also assured that their identity and personal information would not be revealed in any data/information arising from the research study. Interviews were audio recorded with consent using a password-protected and encrypted audio recorder. Once the audio recordings were transcribed (and pseudonymized), these were then erased, retaining data only in an anonymous format.

Given that initially focus group interviews, lasting 90 minutes each, were to be conducted, a token of appreciation, a €10 voucher, was determined appropriate to acknowledge the participants’ time and inconvenience during such a period, and was thus mentioned in the participants’ information and consent documents. As some participants were already recruited to the study (but not interviewed) before the change in data collection method, all participants were offered this token of appreciation on completion of their interview.

### Data analysis

The participants’ characteristics were reported using frequency percentages and median values. All audio recordings were transcribed verbatim with anonymization and imported into NVIVO (version 1.5.1). To maintain the validity and reliability of the acquired data, the transcripts in Maltese were not translated^27^. As recommended by Chen and Boore^28^, analysis was conducted in the original language (Maltese or English), generating categories in the source language and then translating all identified themes (and matching phrases) into English.

All transcripts were analyzed by JG using applied thematic analysis, a rigorous, inductive method for identifying themes from text with the aim of presenting the meanings of the study participants as accurately and comprehensively as possible^27^. The identified themes were then organized according to the different components of the IMB model^17^ and also illustrated in a figure format.

Several strategies were adopted to enhance rigor. A draft coding scheme was developed by JG based on the initial four transcripts analyzed. The coding scheme and the codes were reviewed by the other authors and revised accordingly. The coding scheme was also reviewed after a couple of weeks of analysis, refining, and renaming themes/codes to reflect the meanings of the relevant datasets, enhancing reliability^27^. Generated themes and sub-themes were supported by excerpts from the original participant data; English translations of quotes in Maltese were provided. Additionally, the methods undertaken and data analysis processes were documented and presented so that this study can be replicated^27^.

## RESULTS

### Participant characteristics

The sample included ten former and ten current smokers. The participants’ characteristics, including their diabetes and smoking profiles, are outlined in Table 1. Most participants were middle-aged males with type 2 diabetes. They had at least a secondary level of education and were in employment. Nine participants reported having diabetic complication(s), with five having ischemic heart problems associated with their diabetes status. All smokers smoked daily, smoking on average 16 cigarettes per day. Six current smokers were motivated to quit smoking. However, only two were planning to quit within the next month. All former smokers were previously daily smokers.

**Table 1:** Characteristics of the interviewees recruited from the diabetes care clinics within the two main acute public hospitals in Malta in 2021 (N=20)

*Characteristics*	*n*	*%*
**Demographics**		
**Gender**		
Male	14	70
Female	6	30
**Median age** (years)	51	
**Education level**		
Primary level	2	10
Secondary level	8	40
Post-secondary level	3	15
Vocational training	2	10
Diploma	3	15
Degree	2	10
**Employment status**		
Student	2	10
Employed	10	50
Unemployed (disability)	1	5
House duties	2	10
Retired	5	25
**Diabetes profile**		
**Diabetes type**		
Type 1	6	30
Type 2	14	70
**Median age at diagnosis** (years)	32.50	
**Diabetes complications**		
No	11	55
Yes	9	45
**Smoking profile**		
**Smoking status**		
Median age starting smoking (years)	15	
Median number of years since quitting amongst former smokers	2.21	
Median number of cigarettes/day amongst smokers^a^	16	
**Motivation to quit amongst smokers**		
Planning to quit in <1 month	2	20
Planning to quit in ≥1 month	4	40
Not motivated to quit	4	40

a Excluding one participant who smoked five cigarillos per day.

### Main findings based on the IMB model

**Figure 1:**
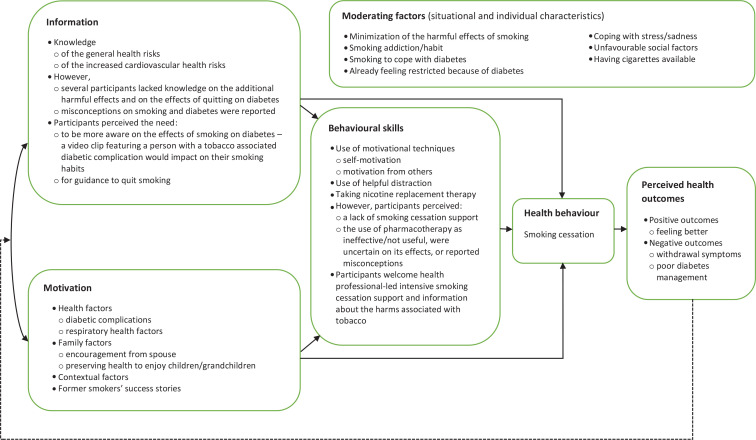
Main findings based on the IMB model, insights from interviews with participants recruited from diabetes care clinics in Malta in 2021 (N=20)

The main findings of this study are organized according to the IMB model^17^. Figure 1 outlines the identified diabetes-specific IMB strengths and deficits, and the identified moderators.

*Information*

Knowledge of smoking, smoking cessation, and diabetes

All participants, except one, were aware of the general health risks associated with smoking, mostly referring to respiratory and cardiovascular health problems. The majority (n=14) were also aware of the increased health risks for those who have diabetes, highlighting the increased cardiovascular health risks (n=9). Conversely, one former smoker and five current smokers stated that they were not aware of any additional health risks.

Few participants (n=7) were aware of the positive effects of quitting on diabetes. Six participants just referred to having overall better health, while four and two participants understood that they would have better blood circulation and controlled diabetes, respectively. On the other hand, three former smokers and six current smokers were unaware of the effects of quitting on diabetes. Furthermore, five current smokers (CSs) and two former smokers (FCs) believed that smoking helps in diabetes management, such as in glucose control (n=5):

*‘When I smoke a cigarette, my blood sugar doesn’t go up … and then if I don't smoke that cigarette, I would feel my blood sugar going up.’* (CS 3, male, translated quote)

and to control overeating (n=3):

*‘The cigarette keeps me from picking up on other things ... It’s as if it makes me feel full, (grinning) I have eaten, I took a cigarette, and that’s it!’* (CS2, female, translated quote).

Three current smokers did not believe that smoking affects diabetes management:

*‘At first, I was worried about how it would affect me. But then I kept checking (blood glucose) before and after to see how it would affect me, but I didn’t see any particular issues, so.’* (CS4, male)

Perceived relevant information to support smoking cessation

Most participants (n=10) perceived the need for more awareness of the effects of smoking on diabetes to encourage smoking cessation. In addition, two participants perceived the need for guidance to quit smoking. Conversely, five participants stated that they would not seek any information.

Views on the provision of information on tobacco-associated diabetic complications to influence smoking habits

Eighteen participants perceived that an increase in awareness of tobacco-associated diabetic complications would impact their smoking, out of concern (n=15):

*‘Maybe these are things which I don’t know about … That is, I am doing some harm because I am diabetic.’* (CS7, male, translated quote)

and out of fear (n=3):

*‘Because I'm afraid of the future … I have diabetes, I mean, I don’t want to add to any consequences.’* (CS2, female, translated quote)

Ten participants also perceived that the use of visual images of tobacco-associated diabetic complications would be effective out of concern or fear. However, the remaining participants (five current and five former smokers) did not agree because they were not affected by the warning images on tobacco products (n=4):

*‘Since those did not affect me, I don’t think other illnesses would affect me.’* (CS10, male, translated quote)

or because they had low perceived susceptibility to such complications (n=2):

*‘Mostly because I see them as something that won't happen to me (laughing).’* (CS4, male)

Another four participants would simply avoid such messages:

*‘To tell you the truth, I would try not to see them.’* (CS6, female, translated quote)

Conversely, more participants (n=17) perceived that watching a video clip featuring a person who had stopped smoking because of a tobacco-related diabetic complication would impact their smoking, mainly out of concern (n=5), fear (n=4), or because it is a real story (n=5):

*‘They’re going to tell you what they went through.’* (CS2, female, translated quote)

Three participants added that it would be easier to follow and understand, and two participants stated that it would be inspiring.

*Motivation*

Various motivational factors to stop smoking or avoid a relapse were reported (Table 2). Most participants (n=16) mentioned health factors as motivators, in particular, prevention of diabetic complications (n=13). Other participants referred to family factors (n=10), contextual factors (n=4), and former smokers’ success stories (n=2) (Table 2). On being prompted, only eight former and three current smokers stated that having diabetes was a motivator to quit smoking.

**Table 2:** Reported motivational factors to quit smoking or to avoid relapsing, interview findings (N=20)

*Themes and sub-themes*	*Quotes*	*n*
**Health factors**		16
**Diabetic complications**		13
Experiencing diabetic complications	*‘The situation from my feet! That’s what they make me stop.’* (FS3, male)	7
Knowledge of possible diabetic complications	*‘Because even because of diabetes, it would affect a lot of things.’* (FS6, female, translated quote)	6
**Respiratory health factors**	*‘The cigarette started to affect my breathing.’* (FS6, female, translated quote)	5
**Family factors**		10
Encouragement from spouse	*‘My wife (laughing) ... every second “Stop stop stop” … It’s one of the most motivations I have.’* (CS9, male)	5
Preserving health to enjoy children and grandchildren	*‘I have a child ... despite having diabetes I wish I could at least enjoy him.’* (CS5, female, translated quote)	5
**Contextual factors**		4
Public smoking restrictions	*‘I was going to go abroad … I said, “It’s going to take long to smoke having to spend three hours on a plane”.’* (CS10, male, translated quote)	2
Getting a home loan	*‘For taking a loan, you know, nowadays you can’t buy a house without taking a loan, so emm, you know.’* (CS9, male, translated quote)	2
**Former smokers’ success stories**	*‘I think the most encouraging thing is the experiences of others, emm … those who used to smoke and managed to quit.’* (CS2, female, translated quote)	2

FS: former smoker. CS: current smoker.

*Behavioral skills*

Smoking cessation facilitators

Various facilitators for smoking cessation were reported (Table 3). The majority (n=11) highlighted the importance of motivational techniques and the usefulness of using helpful distractions (n=7). Three participants remarked on the need to make use of nicotine replacement to quit smoking.

**Table 3:** Reported facilitators to quit smoking, interview findings (N=20)

*Themes and sub-themes*	*Quotes*	*n*
**Motivational techniques**		11
**Self-motivation**		9
Self-motivating talk	*‘I keep in mind that ... “tomorrow is the third day without cigarettes, come on let’s try further!”.’* (CS2, female, translated quote)	6
Thinking about experienced health complications	*‘I kept looking at my chest and picturing a spring inside (chuckling).’* (FS5, male)	4
**Motivation from others**	*‘My children ... used to tell me, “come on be brave, come on, how good you are, come on!”.’* (FS10, female, translated quote)	4
**Helpful distraction**		7
Action distraction	*‘Not sitting down, you know. Making that one, making some DIY, go to the field, go for a walk.’* (CS9, male)	4
Mouth distraction	*‘emm you know I told you these Nicotine inhalers that I bought, so I still have one without nicotine and anything and I just put it in my mouth and that’s it.’* (FS1, male)	2
Thinking distraction	*‘When I tried to quit smoking, I made my mind busy in another thing.’* (CS1, male)	2
**Taking nicotine replacement**	*‘I think a substitute for something, that when you take it you would want this to make you feel calm, because that’s what it is, you feel anxious when you crave a cigarette.’* (CS3, male, translated quote)	3

FS: former smoker. CS: current smoker.

Attitudes towards the use of pharmacotherapy for smoking cessation

Six participants reported positive attitudes towards the use of pharmacotherapy, perceiving it as effective out of the personal experience or of others (n=3), and helpful if lacking willpower (n=3). Conversely, the use of pharmacotherapy was perceived as ineffective by nine participants:

*‘I don't really believe they work … didn't seem they worked on me.’* (CS3, male, translated quote)

Furthermore, four participants did not perceive its need, stating that having willpower is enough:

*‘I never took these things. I quit smoking with my own willpower.’* (FS8, female, translated quote)

In addition, two participants were uncertain about the effect of using pharmacotherapy:

‘I’ve tried the nicotine inhalers … I don’t know if they helped or not.’ (FS1, male)

Three participants were concerned about the possible health consequences of using pharmacotherapy:

*‘Because, for example, I heard … that the patches are harmful.’* (CS6, female, translated quote)

Nonetheless, six current and four former smokers stated that they would consider the use of pharmacotherapy for smoking cessation.

Attitudes towards health professional smoking cessation support

Several participants (n=7) highlighted that there is a lack of smoking cessation advice/support for those who have diabetes:

*‘Whenever I used to ask someone (about the health risks) … they always gave me a generic response of “Smoking is bad” which I just throw out of the window.’* (CS4, male)

Most participants (n=16) welcomed the provision of health professional support for smoking cessation, mainly for providing guidance on how to quit (n=7) and informing them about tobacco-associated harm (n=6). The participants suggested that smoking cessation support should be of an intensive nature, consisting of sessions of half an hour to one hour (n=12), and provided frequently, such as once or twice a week (n=9), over a median value of six weeks.

On the other hand, three participants claimed that they would not seek health professional support to quit smoking, while one participant (FS5) did not hold an opinion on the provision of smoking cessation support.

*Moderators*

Several barriers and challenges that could impact directly on achieving or maintaining abstinence or indirectly by influencing the IMB model constructs or their relationships, were identified (Table 4). Fourteen participants reported experiencing withdrawal symptoms on quitting smoking, particularly nervousness. Nine participants highlighted the smoking habit/addiction. Having diabetes was remarked as a challenge (n=7), mostly in maintaining diabetes management or quitting. Smoking was also found to help participants cope with stress/sadness (n=6).

**Table 4:** Reported barriers and challenges to quit smoking, interview findings (N=20)

*Themes and sub-themes*	*Quotes*	*n*
**Withdrawal symptoms**		14
Nervousness	*‘I started to feel a lot more nervous.’* (CS2, female, translated quote)	11
Sadness	*‘I used to cry. I cried. Do you understand, and sadness, I was sad.’* (FS10, female, translated quote)	3
**Smoking addiction/habit**		9
Smoking addiction	*‘I mean obviously there is also the addiction to the, to the nicotine as well.’* (FS5, male)	5
Smoking habit	*‘Now, if I go out five times by car ... those are five cigarettes, because I only need to smoke while driving.’* (CS3, male, translated quote)	4
**Having diabetes**		7
**Maintaining diabetes management on quitting smoking**		5
Eating more on quitting which does not help diabetes management	*‘You start eating more. And in my position, I can’t start eating more, you know.’* (CS9, male)	5
Loosing glucose control on quitting	*‘The difficulties were ... I started to lose sugar control.’* (FS2, female, translated quote)	4
**Smoking to cope with having diabetes**	*‘At that time it was as if it was a taboo to be diabetic, that is, I used to smoke a lot of cigarettes to cope, so to speak.’* (FS7, male, translated quote)	3
**Already feeling restricted because of diabetes**	*‘Maybe I can’t quit or I’m not interested in quitting, emm because we are restricted in a lot of things, that is, we refrain from taking a dessert after eating, we refrain from alcohol because it raises the blood sugar.’* (CS2, female, translated quote)	2
**Coping with sadness/stress**		6
Coping with sadness	*’I’d rather say, “I smoked a cigarette”, than fall into a depression or so, I would say, “a cigarette is enough, I don’t need anything else!”.’* (CS5, female, translated quote)	4
Coping with stress	*‘Yes, yes, stress, stress. Yes, the cigarette used to calm me down when in stress.’* (FS10, female, translated quote)	2
**Unfavorable social factors**		4
Family members or friends who smoke	*‘I had some friends that smoked as well ... which made it a bit harder.’* (FS1, male)	2
Lack of support	*‘But then it was hard, I think, because I was on my own.’* (FS2, female, translated quote)	2
**Having cigarettes available**	*‘As soon as you buy a packet … you know you will eventually smoke them, so.’* (FS9, male, translated quote)	2

FS: former smoker. CS: current smoker.

In addition, three current smokers attempted to downplay the harmful effects of smoking, undervaluing smoking cessation:

*‘It’s like when someone, for example, tells you, “Listen, stop smoking for your lungs and because of cancer”, but I also know a lot of people who died of cancer and were the healthiest ever.’* (CS2, female, translated quote)

Conversely, four former smokers remarked feeling better about quitting smoking, which encouraged them to remain abstinent.

## DISCUSSION

Despite being aware of the general smoking health risks and the additional risks for individuals with diabetes, the participants still lacked knowledge of the association between smoking, smoking cessation, and diabetes. As in previous studies carried out amongst individuals with type 2^12,13^, and type 1 diabetes^11^, this study’s participants also lacked accurate information, reporting misconceptions about smoking and diabetes.

Nonetheless, as was found in the study of Abu Ghazaleh et al.^11^, the need for more awareness of the effects of smoking on diabetes to support smoking cessation, was expressed by the study participants. While the use of visual images of tobacco-associated diabetic complications has been recommended to raise awareness of such complications for encouraging cessation^23^, this study suggests otherwise, as the participants had mixed feelings about this. Noar et al.^29^, in fact, suggest caution in using tobacco pictorial warnings, as these can also encourage denial or avoidance of such messages. Conversely, the participants were more receptive to the use of video messages featuring former smokers who experienced tobacco-associated diabetic complications to convey such information. The use of such video messages as part of a mass media campaign has been found to increase awareness of tobacco-related harm, quit attempts, and smoking cessation efforts amongst the general population^30,31^. Given these positive findings, future research should investigate the use of such video messages as an educational tool, part of a smoking cessation intervention for individuals with diabetes.

Similar to previous literature^11-13^, most participants identified health as their primary motivator to quit smoking and remain abstinent. As was observed in the study of Georges et al.^12^, only half of the participants stated that having diabetes was a motivator to quit smoking. This suggests further that some participants did not believe that smoking impacted their diabetes management.

Most of the mentioned facilitators or skills for smoking cessation (such as increased health awareness, family support, and helpful distractions) were also identified in previous studies^11,13^. As was found in the literature^11,13^, the participants in this study also identified the need for health professional support to quit smoking. These suggested that this should be intensive, in line with Grech et al.^23^ recommendations. Given the identified lack of smoking cessation support for those with diabetes, the provision of intensive smoking cessation support as part of diabetes management is thus recommended.

Despite the promising use of pharmacotherapy for smoking cessation among individuals with diabetes^22,32^, only half of the participants in this study were in favor of using it. In addition, some participants held negative attitudes or had misconceptions about using pharmacotherapy. This warrants the need to provide more information on the benefits and use of pharmacotherapy for smoking cessation to target any negative attitudes and misconceptions.

As in previous literature^11-13^, several barriers and challenges to quitting or negative moderators to behavior change, such as the smoking habit/addiction or experiencing withdrawal symptoms on quitting, were identified by the study participants. Such challenges in quitting re-confirm the need for health professional support for the identification of high-risk situations of smoking and the generation of problem-solving strategies and the use of nicotine replacement therapy for managing nicotine addiction and withdrawal symptoms. Having diabetes was also reported as a challenge in previous literature^11-13^, in particular, because of possible weight gain or glycemic imbalance. This confirms the need for tailored smoking cessation support for those who have diabetes, presenting an opportunity to introduce smoking cessation support as part of local diabetes education efforts.

Strengths and limitations

Guided by the IMB model, this study helped to identify the unique needs of individuals with type 1 and type 2 diabetes to quit smoking, for the future development of a tailored smoking cessation intervention. As shown in Figure 1, this research identified the diabetes-specific IMB strengths that can be capitalized on, and any deficits that need to be addressed, when designing a smoking cessation intervention^17^. Furthermore, as suggested by Fisher et al.^17^, this study also explored any moderating factors that can influence smoking cessation and abstinence.

Healthcare interventions are very much dependent on patient involvement and their attitudes to them^14^. Hence, this study also explored the participants’ views of the features of smoking cessation interventions, previously identified in a scoping and a systematic review, as showing promise in use with persons with diabetes^22,23^. This study validated the following recommendations: raising awareness of the effects of smoking on diabetes by showing video messages featuring former smokers’ true stories of suffering from smoking-related diseases, and the provision of intensive smoking cessation support.

In this study, the use of purposive sampling ensured adequate representation by gender, age, education level and employment status, and different diabetes and smoking profiles. However, none of the identified participants smoked or used to smoke on an occasional (weekly) basis. Occasional smokers may have different needs and preferences than those identified in this study. Another limitation of this study was that focus group interviews could not take place as previously explained. Nonetheless, the use of phone interviews still provided an in-depth understanding of the participant’s needs and preferences, successfully achieving the aim of the study.

## CONCLUSIONS

Guided by the IMB model, this study helped to identify the unique needs of individuals with type 1 and type 2 diabetes to quit smoking, presenting practice and research recommendations. The study’s findings emphasize the need for more awareness efforts on the effects of smoking on diabetes to encourage cessation. Using video messages that showcase the true stories of former smokers with diabetes who have experienced smoking-related health issues, may have an impact on smoking cessation. Hence, future research should investigate the use of such video messages as an educational tool and as a part of a smoking cessation intervention for individuals with diabetes. Considering the perceived lack of tailored smoking cessation support for those with diabetes and the reported diabetes-specific challenges and barriers to quitting smoking, the provision of intensive smoking cessation support as an integral part of diabetes management is also recommended.

## Data Availability

The data supporting this research are available from the authors upon reasonable request.
